# Removal method of a Supera interwoven stent invaginated during its implantation in endovascular procedure: a case report

**DOI:** 10.1186/s42155-024-00449-3

**Published:** 2024-04-11

**Authors:** Tasuku Kozasa, Masahiko Fujihara, Tomofumi Tsukizawa, Yuko Yazu, Naoko Abe, Ryoki Doami, Yoshiaki Yokoi

**Affiliations:** 1 Department of Cardiology, Kishiwada Tokushukai Hospital, 4-27-1, Kamoricho, Kishiwada-City Osaka, 596-8522 Japan; 2 Department of Medicine and Biosystemic Science, Kyushu University Graduate School of Medical Sciences, Fukuoka, Japan; 3 Department of Clinical Engineering, Kishiwada Tokushukai Hospital, Osaka, Japan

**Keywords:** Interwoven stent, Invagination, Endovascular treatment, Peripheral artery disease, Complication

## Abstract

**Background:**

Supera interwoven stents (IWS) have a unique interwoven structure; thus, precise stent placement can be challenging as they are prone to elongation, shortening, and invagination. Particularly, invagination limits long-term patency. This proposed method aims to remove invaginated IWS.

**Case presentation:**

A 70-year-old man presented with intermittent claudication in his left lower limb. Endovascular therapy was conventionally performed, and a 5.5 × 40 mm IWS was placed after balloon dilatation; however, invagination occurred. The invaginated IWS was successfully removed by a threading 0.014" wire through the outside of the stent strut, and a snare catheter was used to hold it in place from the inside. Then, while still in place, the 0.014" wire and snare catheter were driven into the guiding sheath.

**Conclusions:**

This practical and easy approach to remove invaginated IWS from the body relies on the particular structural characteristics.

**Supplementary Information:**

The online version contains supplementary material available at 10.1186/s42155-024-00449-3.

## Background

Although the Supera interwoven stent (IWS; Abbott, Abbott Park, Illinois) is a bare metal stent without drug technology, its unique interwoven structure with superior compression resistance, flexibility, and conformability makes it suitable for femoropopliteal artery diseases, particularly in cases of severe calcification thrombotic lesions and joint movements. Many reports have demonstrated the safety and long-term patency of the IWS [1–3]. However, precise stent placement can be challenging because of its unique deployment system [4], which is prone to challenging drawbacks, including elongation, shortening, and invagination. In recent years, studies have reported that such invagination limits long-term patency, which is one of the events that should be avoided [5–7]. Often, inadequate stenting cases occur in real-world practice, among which invagination is reported to limit long-term patency [8]. This may be because invaginated stents do not sufficiently spread the stent and lesion, or when the stent strut becomes spider-webbed, resulting in thrombotic restenosis due to the lack of vessel wall attachment. Aggressive vessel preparation before stent placement is considered necessary for appropriate placement of the Supera stent to avoid invagination. In this case, the thrombotic occlusion may have been caused by inadequate predilation of the stent because of concerns about distal embolization. Herein, we describe a successful removal procedure of the IWS from the superficial femoral artery (SFA) following its invagination into the lesion during implantation.

## Case presentation

**Fig. 1:**
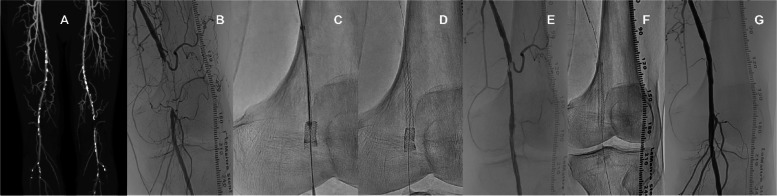
Process of endovascular therapy. **A** Computed tomography angiography image showing occlusion of the left superficial femoral artery distal to the proximal popliteal artery. **B** Basic angiography of the left limb. **C** Invagination occurred during stent deployment. **D** Implanted invaginated stent unavoidably. **E** Angiography for insufficient stent dilation and proximal thrombus clusters. **F** Another new IWS of the same size was accurately placed. **G** Final angiography after stent reimplantation

**Fig. 2:**
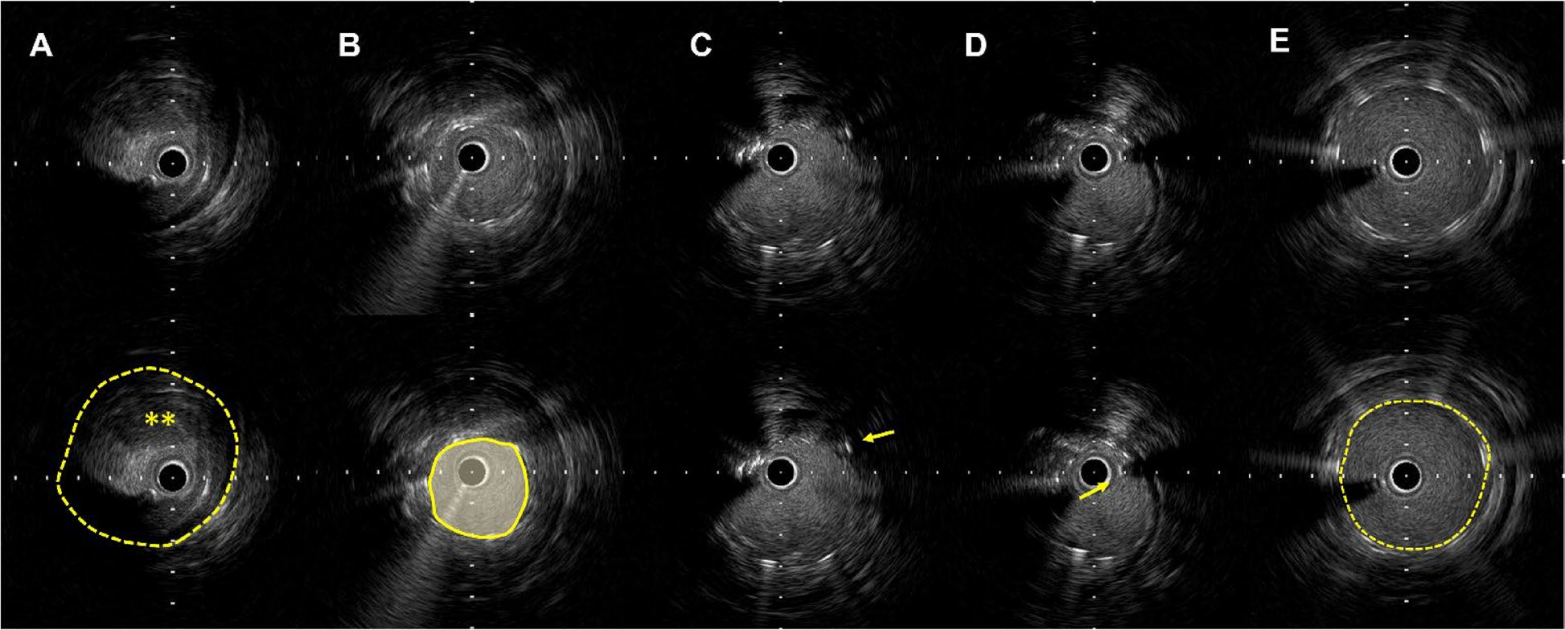
IVUS image. **A** Baseline IVUS findings: thrombus present (**). **B** Post-invaginated stent findings: insufficient dilation (3.4 × 2.9 mm). **C** Wire cross from outside stent strut (yellow arrow). **D** Check wire crossings in the stent. **E** IVUS after treatment, confirming sufficient dilation (5.2 × 5.1 mm)IVUS, intravascular ultrasound

A 70-year-old man with Rutherford category 3 of intermittent claudication in the lower left limb was referred to our institution. He had been symptomatic for ~ 2 years and been treated conservatively; however, his symptoms had rapidly worsened over the past month. The patient was a past smoker and had diabetes and dyslipidemia. The ankle–brachial index (ABI) on the left side dropped to 0.43. Enhanced computed tomography angiography showed calcification stenosis (PACSS grade 2) and short-segment occlusion from the SFA to the proximal popliteal artery (Fig. 1A). Initially, endovascular therapy was conventionally performed, with contralateral crossover access using a 6-FR guiding sheath (Destination, Terumo, Tokyo, Japan) (Fig. 1B). After the passage of the 0.014" guidewire (Command 0.014, Abbott), the lesion characteristics and vessel size were confirmed by intravascular ultrasound (IVUS; Altaview, Terumo Tokyo, Japan). Since the occlusion was poorly calcified and mainly a chronic thrombus component, balloon dilation adapted to the vessel diameter was considered a risk for distal embolization (Fig. 2A). Therefore, a 4-mm small-diameter balloon (Oceanus 4 × 40 mm, i-vascular, Barcelona, Spain), was dilated, no filter device was used in this situation. and the guidewire size was changed to 0.018" (V-18, Boston Scientific, Marlborough, NA, USA). Afterward, we attempted the placement of a 5.5 × 40 mm Supera stent (Abbott).

However, the stent was implanted without sufficient expansion (Fig. 2B), and invagination occurred (Fig. 1 C–E), due to misdeployment. Because long-term patency was not expected and the risk of thrombo-occlusion was considered high, we proceeded with the removal of the invaginated stent.

**Fig. 3:**
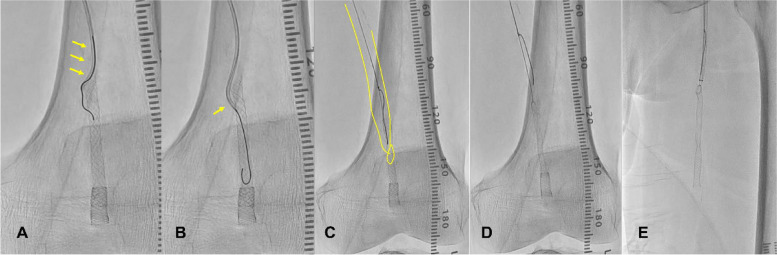
Supera stent removal method. **AB** Using a JR catheter, a wire was passed from the outside of the stent through the strut and into the stent. **C** Snare catheter through the stent and grip the wire from the outside. **D** Slowly pull both wires to remove the stent from the vessel wall. **E** Store everything inside the guiding sheath

**Fig. 4:**
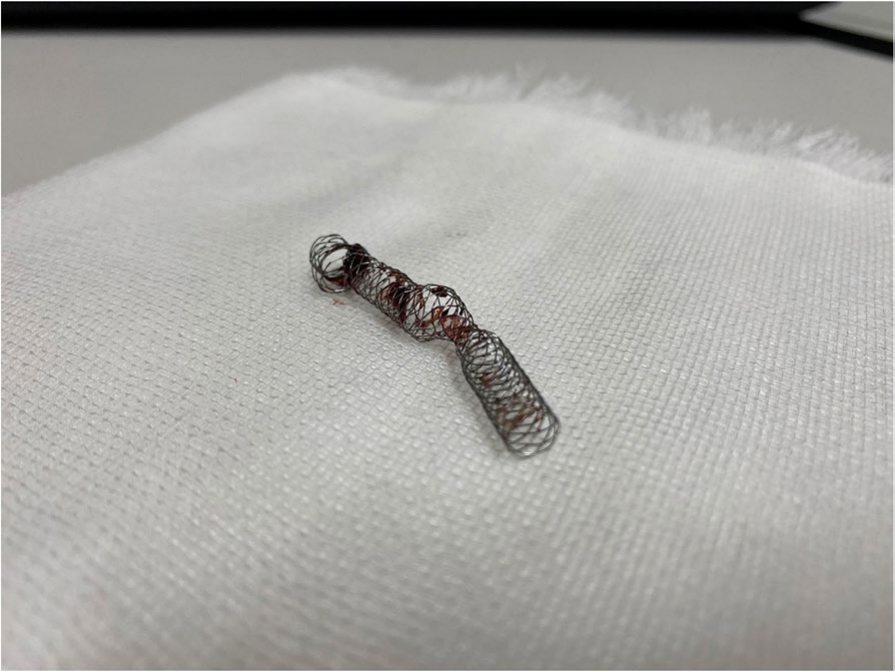
Supera stent removed from the body with thrombus adherence but no stent fracture or breakage

First, the contralateral approach was up to the 7-FR system (Destination 7FR, Terumo). We approached the JR catheter near the invaginated stent and passed a 0.014" wire into the outer side of the stent through the strut (Fig. 3A and B). The wire site was confirmed by IVUS (Fig. 2C and D), and the 0.014 wire was caught using a 7mm gooseneck snare catheter (Amplatz Goose Neck Snares, Medtronic, Santa Rosa, CA, USA) (Fig. 3C) into the inside of the invaginated stent. Then, we withdrew the 0.014" wire and the snare catheter into the guiding sheath (Fig. 3D and E) while still in place. Finally, we successfully removed the Supera from the body (Fig. 4). Another new IWS (5.5 × 40 mm) of the same size was then accurately placed and post-dilated at 5 × 40 mm (Sterling Boston Scientific). Although a nonflow limiting dissection was found in the proximal portion of the stent, sufficient dilation was confirmed, without residual stenosis, and the procedure was completed (Fig. 1F-G). An additional postoperative consideration is that bringing a longer sheath into the proximal portion of the stent would allow safer removal.

Postoperatively, his symptoms improved quickly, and his ABI increased to 1.1 the next day. The stent remained open without restenosis for 3 months.

## Conclusions

In this case report, we described the practical removal procedure of a IWS that was invaginated during its implementation for endovascular treatment. Invaginated Supera stents are reportedly prone to restenosis due to their lumen-occupying structures and thrombus formation. Repeated endovascular treatments are often necessary, but achieving long-term patency remains challenging. Therefore, despite the risks associated with stent retrieval, stent retrieval may be preferable in some situations. Upon immediate implantation, the intima remains uncoated, minimizing vessel damage. However, over the mid- to long-term, the intima becomes coated, making retrieval unfeasible. For this, a 0.014" wire was threaded through the outside of the stent strut, and a snare catheter was used to hold it in place from the inside and successfully remove it from the body. Although the treatment method reported here is relatively simple and easy, it is not feasible with other common nitinol stents. It specifically applies to IWS, owing to its special characteristics, as follows. First, the Supera stent has a chronic outward force lower than that in other stents [9]; thus, it does not adhere to the vessel wall immediately after implantation. It also has high anti-kink properties which reduces the risk of damaging vessels during removal. In addition, the stent is composed of six interwoven nitinol wires, which, when held in one position, enable the pulling of the entire stent while becoming thinner. The case was then re-implanted with a Supera stent; however, other treatment options might be available if the thrombus could be removed along with the Supera. Our findings demonstrate a practical and easy approach to removing invaginated Supera stents are placed in the body, relying on its special structural characteristics; however, this complication should be avoided. Using IVUS to identify the exact location to hook the wire and the snare route may be convenient and nondamaging for proximal vessels. They offer new means to address practical challenges that practitioners face during stent implementation in endovascular treatment.

### Supplementary Information

**Additional file 1. **Procedural steps for removal of invaginated IWS during endovascular treatment.

**Additional file 2. **

## Data Availability

The data are available from the corresponding author upon reasonable. request.

## Abbreviations

IWS: Interwoven stent
SFA: Superficial femoral artery
IVUS: Intravascular ultrasound
