# Supplementary material for: Removal method of a Supera interwoven stent invaginated during its implantation in endovascular procedure: a case report
Source: CVIR Endovasc. 2024 Apr 11;7:36. doi: 10.1186/s42155-024-00449-3 (PMC11009178; doi:10.1186/s42155-024-00449-3)
Supplement: Supplementary file 2 — Additional file 2. [file 42155_2024_449_MOESM2_ESM.pptx]

## Slide 1
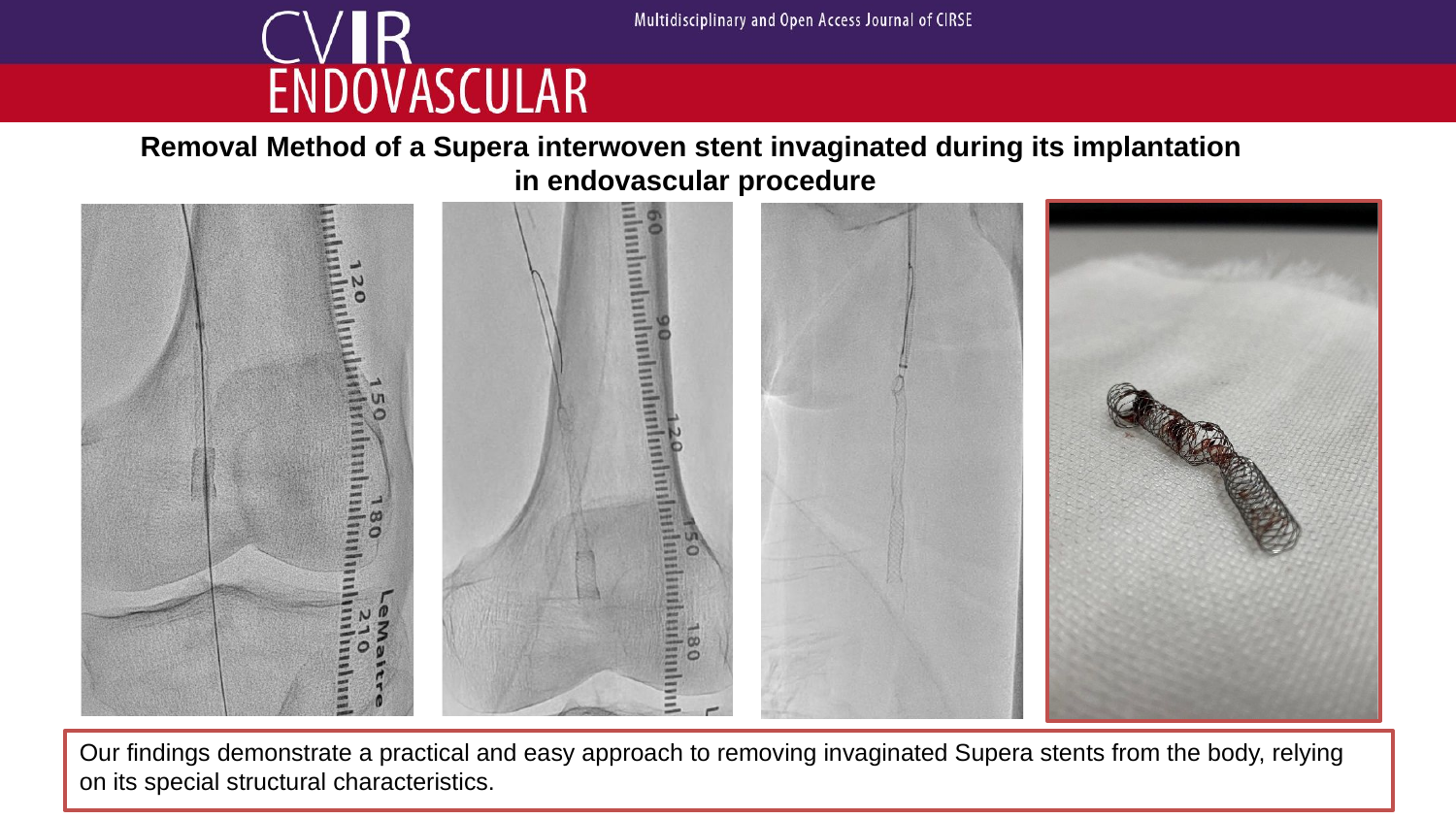

# Removal Method of a Supera interwoven stent invaginated during its implantation in endovascular procedure
Our findings demonstrate a practical and easy approach to removing invaginated Supera stents from the body, relying on its special structural characteristics.
